# Clinician, Youth, and Parent Perspectives on Diabetes Technology Education

**DOI:** 10.1177/26350106251383865

**Published:** 2025-11-17

**Authors:** Brynn E. Marks, Seema Meighan, Dorene F. Balmer, Randi Streisand, Joseph I. Wolfsdorf, Andrea Kelly, Victoria A. Miller

**Affiliations:** Children’s Hospital of Philadelphia, Division of Endocrinology & Diabetes, Philadelphia, Pennsylvania; Department of Pediatrics, University of Pennsylvania Perelman School of Medicine, Philadelphia, Pennsylvania; Children’s Hospital of Philadelphia, Division of Endocrinology & Diabetes, Philadelphia, Pennsylvania; Department of Pediatrics, University of Pennsylvania Perelman School of Medicine, Philadelphia, Pennsylvania; Department of Psychology and Behavioral Health, Children’s National Hospital, Washington, District of Columbia; Division of Endocrinology, Boston Children’s Hospital, Department of Pediatrics, Harvard Medical School, Boston, Massachusetts; Children’s Hospital of Philadelphia, Division of Endocrinology & Diabetes, Philadelphia, Pennsylvania; Department of Pediatrics, University of Pennsylvania Perelman School of Medicine, Philadelphia, Pennsylvania; Department of Pediatrics, University of Pennsylvania Perelman School of Medicine, Philadelphia, Pennsylvania; Children’s Hospital of Philadelphia, Division of Adolescent Medicine, Philadelphia, Pennsylvania

## Abstract

**Purpose:**

The purpose of this study was to explore the educational experiences of youth with type 1 diabetes (T1D), their parents, and clinicians when initiating continuous glucose monitoring (CGM) and insulin pumps.

**Methods:**

Twenty parent-child dyads with T1D ≥6 months and ≥1 month CGM and insulin pump use were eligible to participate in semistructured dyadic interviews. Purposive sampling was used to recruit youth with a range of A1C levels and to overrepresent dyads from minoritized backgrounds. Eight diabetes clinicians with ≥1 year of experience participated in individual interviews using a parallel interview guide. A subset of interviews was double-coded, and thematic analysis was used to generate themes.

**Results:**

Poor internet connections, distractions in the home, and small screens made in-person education the preferred modality for dyads and clinicians due to the physical skills required when learning to use these devices. Structured education addressing essential topics was constrained by allotted appointment times and thus (1) often overlooked cognitive and emotional burdens of diabetes technology education and (2) insufficiently accounted for individual learning pace and capacity. Real-world experiential learning supported by the clinical team through telemedicine, phone calls, and electronic medical record messaging was often used to fill the gaps of structured education.

**Conclusions:**

Both clinicians and parent-child dyads initiating CGM and automated insulin delivery expressed a preference for in-person education. Although experiential learning can supplement important concepts not adequately addressed during structured education, relying solely on this approach may unintentionally omit crucial concepts. Educational strategies are needed to overcome information overload and support families in diabetes self-management.

Attaining glycemic targets for people with type 1 diabetes (T1D) requires lifelong adherence to glucose monitoring and insulin therapy along with real-time adjustments to insulin delivery to account for variations in diet and activity. Continuous glucose monitoring (CGM) is a wearable technology that measures glucose levels every 1 to 5 minutes throughout the day and sends alerts for hypoglycemia and hyperglycemia to the user. Another technology, insulin pump therapy, provides a continuous subcutaneous infusion of rapid-acting insulin that more closely simulates endogenous insulin secretion. CGM data can be shared with an automated insulin delivery (AID) system that uses an algorithm to automatically increase or decrease insulin delivery through an insulin pump. These advances in diabetes technologies are standard of care for youth with diabetes because of their ability to improve metabolic outcomes while decreasing the burden of care for youth with T1D and their caregivers.^1-6^

Learning to effectively and independently use these diabetes devices can be challenging. To achieve optimal benefits, effective use of CGM and AID requires extensive user interaction,^7,8^ which must be supported by in-depth, structured self-management education for youth with T1D and their families.^9^ As part of structured education, clinicians must assess baseline knowledge and experiences while also being cognizant of the cognitive load created by the material being taught. Cognitive load theory recognizes that the mental demands of an educational experience are related to both the intrinsic, extrinsic, and germane loads.^10^ Although the inherent challenges of the information pertaining to CGM and AID cannot be altered (intrinsic load), clinicians can optimize how pertinent information is presented (extrinsic load) to support families in synthesizing the information and developing problem-solving skills (germane load). Furthermore, the COVID-19 pandemic accelerated the transition to telemedicine, and many families continue to have access to telemedicine for diabetes technology education.

Although quantitative outcomes from telemedicine CGM and AID education have been assessed,^11-14^ the nuanced experiences of youth, parents, and clinicians remain underexplored.^15^ As diabetes technologies continue to rapidly evolve, understanding the experiences of clinicians and parent-child dyads is essential for adapting educational practices to meet the evolving needs of all stakeholders and to support youth in attaining glycemic targets. The purpose of this qualitative study was to explore the educational experiences and preferences of youth with T1D, their parents, and T1D clinicians when initiating CGM and AID.

## Methods

This study follows the Consolidated Criteria for Reporting Qualitative Research reporting guideline to promote transparency and quality of the research process^16^ and was approved by the Children’s Hospital of Philadelphia (CHOP) Institutional Review Board (IRB 22-019791). Informed consent was obtained from all participants ≥18 years, and verbal assent and parental permission were obtained for youth <18 years.

### Paradigmatic Stance and Reflexivity

An interpretivist stance was used in this study, meaning that to understand participants’ experiences, the authors needed to explore how participants made sense of learning to use CGM and AID. The authors acknowledge their role as interviewers (BEM, SM) impacted participants’ synthesis of these experiences. One interviewer (BEM) is a pediatric endocrinologist who has lived with T1D for 35 years and uses an AID system. BEM earned a master’s degree in education and directs the Diabetes Technology Program at CHOP. Another interviewer (SM) is a doctorally prepared nurse with over 30 years of experience in diabetes care. Neither interviewer was the treating clinician for any of the parent-child dyads, and neither was a supervisor of the participating diabetes clinicians.

### Study Design and Population

Parent-child dyads and diabetes clinicians at the Diabetes Center at CHOP were eligible to participate in semistructured interviews. Youth ages 8 to 12 years with T1D for ≥6 months and ≥1 month experience using CGM and AID were eligible to participate together with the parent most involved in T1D care. Clinical dashboards were used to identify eligible youth, who were then contacted via the electronic medical record (EMR) or in person at diabetes appointments. Purposive sampling was used to recruit youth with a range of A1C values and to overrepresent dyads from historically minoritized backgrounds to better understand factors that could improve technology uptake in this group. Clinicians at the CHOP Diabetes Center, including diabetes care and education specialists, nurse practitioners, and psychologists, were eligible to participate if they were employed in their current role ≥1 year. Clinicians with experience in diabetes technology education at both the main campus and satellite clinic locations were prioritized. Eligible clinicians were invited to participate via email.

### Data Collection

Sociodemographic information and pertinent diabetes medical history were collected from youth and caregivers using a REDCap survey.^17,18^ Surveys were completed before the interviews. REDCap was also used to gather sociodemographic information and diabetes clinical experience for the clinicians.

### Interviews

Recognizing that the goal of diabetes technology education is to support families experiencing T1D in effective and independent self-management, the Association of Diabetes Care and Education Specialists framework for self-care behaviors informed the development of interview guides for dyads and clinicians.^19^

This framework emphasizes the importance of taking a person-centered approach to support effective behavior change as part of diabetes self-management, with particular emphasis on problem-solving skills and reducing the cognitive load, which were topics of focus in the interviews. The guide included open-ended questions pertaining to experiences with diabetes technology, experiences with CGM education, experiences with insulin pump education, and educational priorities (Table 1). Follow-up prompts and spontaneous probing questions were included when necessary to gain further insights.^20,21^ Interviews with clinicians followed an interview guide parallel to the one used with the dyads (Table 2).

**Table 1.** Parent-Child Dyad Semistructured Interview Guide

**(1) T1D technology initiation** We would like to start by understanding more about your family’s experience with diabetes technologies. a. What made you and your family interested in using a CGM and insulin pump? **(2) Experiences with CGM education** Next, we would like to hear about your experiences using the CGM and with CGM education. a. What CGM does [child] use, and how long have you been using it for? b. How did you first learn to use [child’s] CGM? i. Was this session virtual or in person? ii. Was this session 1 on 1 or in a group setting? iii. At CHOP or somewhere else? iv. What was helpful about that learning experience? c. In your family, who takes the lead in using the CGM? i. Who puts the CGM on? ii. Who responds to the CGM alerts and alarms? iii. Who, if anyone, reviews the CGM data? iv. Tell me about your experience using the CGM follow feature to keep an eye on [child]’s CGM numbers when he/she is not with you? d. What have you found most helpful in gaining confidence with CGM use? e. Tell me about some behavioral challenges you have encountered related to CGM use? a. What is it like to see so many glucose values and trend arrows? b. What has your experience been with CGM alerts and alarms? c. How comfortable do you feel adjusting insulin doses based on patterns you see on the CGM? d. How do you troubleshoot when there are problems with the CGM? e. Please share an example of how you have handled conflict related to CGM use with your child? f. What things do you wish you had learned sooner after starting CGM use? g. What areas of CGM use would you like to learn more about to help you with [child]’s diabetes management? **(3) Experiences with insulin pump education** Next, we would like to hear about your experiences using the insulin pump and insulin pump education. a. What pump does [child] use, and how long have you been using it for? b. How did you and your family first learn to use [child]’s current insulin pump? a. Was this session virtual or in person? b. Was this session 1 on 1 or in a group setting? c. At CHOP or somewhere else? d. What was helpful about that experience? c. In your family, who takes the lead in using the insulin pump? a. Who fills the insulin pump with insulin? b. Who puts the pump sites in? c. If using AID: Who connects the pump and CGM? d. Who responds to the pump alerts and alarms? e. How do you troubleshoot when there are problems with the insulin pump? f. Who, if anyone, reviews the insulin pump reports? d. What have you found most helpful in gaining confidence with insulin pump use? e. What behavioral challenges do you encounter related to insulin pump use? a. What has your experience been with insulin pump alerts and alarms? b. How comfortable do you feel adjusting insulin doses in the pump? c. Please share an example of how you have handled conflict related to insulin pump use with your child. f. What things do you wish you had learned sooner after starting [child]’s insulin pump? g. What areas of insulin pump use would you like to learn more about to help you with [child]’s diabetes management? **(4) Is there anything else that you would like our team to know?** **(5) *Have child leave room*:** [Child], thank you for sharing your thoughts with us! We have just a few more questions for your [caregiver]. You can head out to [insert space] while we ask your [caregiver] just a few more questions. a. Thank you for your insights on all of those questions. There can be some aspects of diabetes management that might be difficult to discuss with [child] in the room. Is there anything else that you wanted our team to know now that [child] has left?

Abbreviations: AID, automated insulin delivery; CGM, continuous glucose monitor; CHOP, Children’s Hospital of Philadelphia; T1D, type 1 diabetes.

**Table 2.** Clinician Semistructured Interview Guide

**(1) T1D technology initiation** We would like to start by understanding more about your role on the diabetes team. a. What is your role on the CHOP diabetes team? b. In your role, how do you interact with children and parents using CGMs and insulin pumps to manage T1D? **(2) Experience with CGM education** *(select relevant questions based on clinical role)* Next, we would like to hear about your experiences with CGM education. a. Please describe how children and families initially learn to use a CGM at CHOP. b. What happens when families upgrade to newer CGM systems or change to a new system? c. What is most effective about these learning experiences? d. What areas of CGM use require additional education to help families to use this technology to its full potential? e. What behavioral challenges do you encounter related to CGM use? **(3) Experiences with insulin pump education** *(select relevant questions based on clinical role)* Next, we would like to hear about your experiences with insulin pump and AID education. a. Please describe how children and families learn to use insulin pumps and AID systems at CHOP. b. What happens when families upgrade to a newer pump or change to a new system? c. What is most effective about these learning experiences? d. What areas of insulin pump and AID use require additional education to help families to use this technology to its full potential? e. What behavioral challenges do you encounter related to insulin pump and AID use? **(4) Is there anything else that you would like our team to know?**

Abbreviations: AID, automated insulin delivery; CGM, continuous glucose monitor; CHOP, Children’s Hospital of Philadelphia; T1D, type 1 diabetes.

BEM and SM conducted audio-recorded, semistructured interviews via teleconferencing software between January 2023 and August 2023. Median interview duration for youth-parent dyads and clinicians was 52.0 ± 8.1 minutes and 50.8 ± 9.0 minutes, respectively. Field notes were used to capture evolving concepts so that they could be further explored in subsequent interviews.

Interviews were conducted until information power was achieved.^22^ Information power recognizes that the more information contained in the interviews, the lower the number of participants needed. Given the narrow focus of the study, which explores experiences with diabetes technology education in school-age children, and the quality of the dialogue, the experiences shared by 28 participants allowed for a thorough exploration of the topic.

### Data Analysis

Descriptive statistics were used to report demographic and clinical characteristics of the dyads and career experiences of the clinicians. STATA software (version 18.0, StataCorp, LLC, College Station, TX) was used for quantitative data analysis.

Semistructured interview transcripts were deidentified before being uploaded into NVivo qualitative software (Mac, release 1, QSR International, Doncaster, Australia) for coding and data analysis. Data were coded and analyzed in accordance with an inductive approach to thematic analysis.^23^ Two authors (BEM, SM) partnered in the development of inductive, iterative codes. Codes were compiled into a codebook that was used to code all interviews.^24,25^ Twenty percent of interviews were coded by both BEM and SM to ensure dependability of their coding. Coded data were reviewed to develop themes in domains of interest.^26^ The first author summarized the coded data and generated themes based on review of the summaries across all participants. Final themes were reviewed iteratively by team members until a consensus was reached.

## Results

### Participant Characteristics

Twenty parent-child dyads and 8 diabetes clinicians were interviewed. Youth had an average T1D duration of 4.6 ± 2.9 years upon enrollment. Average A1C was 7.6% ± 1.5% (range: 5.4%-10.9%). The group was racially and ethnically diverse, with 30% identifying as non-Hispanic Black and 10% as multiracial or “Other.” Of the 8 diabetes clinicians who participated, 4 (50%) were diabetes educators, 3 (37.5%) were nurse practitioners, and 1 (12.5%) was a diabetes psychologist. One of the clinicians reported personally using AID for T1D management. Two clinicians reported that family members used CGM for diabetes management, but none of the clinicians had a family member using a pump or AID. Additional demographic characteristics are detailed in Table 3.

**Table 3.** Sociodemographic Characteristics of Participants

	Parents (n = 20)	Youth (n = 20)	Clinicians (n = 8)
Age, y	41.3 ± 4.7	10.5 ± 1.4	40.6 ± 8.2
Female sex (%)	16 (80%)	13 (65%)	8 (100%)
Race/ethnicity			
NHW	13 (65%)	12 (60%)	6 (75%)
NHB	6 (30%)	6 (30%)	0
Other	1 (5%)	2 (10%)	2 (25%)
Experience, y	Age at T1D diagnosis: 5.9 ± 2.9 T1D duration: 4.6 ± 2.9 CGM use: 2.8 ± 1.6 Insulin pump use: 0.8 (IQR 0.4, 3.4)		Time in current role: 12 ± 9.6
Duration of diabetes technology use, y	CGM: 2.8 ± 1.6 Pump: 0.8 (0.4, 3.4)		
CGM system	Dexcom G6: 19 (95%) Freestyle Libre 2: 1 (5%)		
Insulin pump or automated insulin delivery system	Tandem Control IQ: 3 (15%) Omnipod 5: 13 (65%) Omnipod Open Loop: 4 (20%) DIY: 1 (5%)		
Glycemia	A1C: 7.6% ± 1.5% CGM time in range (70-180 mg/dL): 53.2% ± 18.6% CGM time above range (>180 mg/dL): 43.4% ± 18.9% CGM time below range (<70 mg/dL): 2.8% (IQR 1.5, 4)		
Clinical role			Certified diabetes care and education specialist: 4 (50%) Nurse practitioner: 3 (37.5%) Psychologist: 1 (12.5%)

Abbreviations: CGM, continuous glucose monitoring; IQR, interquartile range; NHB, non-Hispanic Black; NHW, non-Hispanic White; T1D, type 1 diabetes.

Eighty-five percent (n = 17) of parent-child dyads completed individual CGM training with a CHOP diabetes educator, of whom, 59% (n = 10) completed in-person education, 35% (n = 6) completed telemedicine training, and 1 family completed both in-person and telemedicine education. Against recommendations from their clinical teams, 3 dyads (15%) self-started CGM without completing training with the medical team. Due to the COVID-19 pandemic and institutional practices at the time of insulin pump therapy initiation, 14 (70%) dyads completed pump training through telemedicine, and 6 (30%) were trained in person. All clinicians had experience with providing CGM, insulin pump, and AID education both in person and through telemedicine.

### Interviews

Seven themes emerged in 3 topic areas, which were identified post hoc^26^: (1) in-person versus telemedicine diabetes technology education, (2) information overload, and (3) the importance of experiential learning. The 18 participant quotes included in the article capture the perspectives of 8 of 20 dyads (40%) and 5 of 8 clinicians (63%).

## Topic Area 1: In-Person Versus Telemedicine Diabetes Technology Education

**Figure 1.**
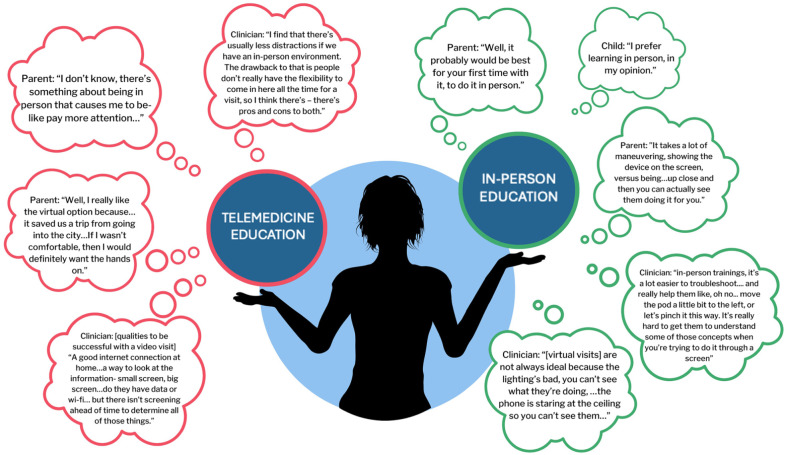
Perceived benefits and challenges to telemedicine and in-person continuous glucose monitoring, insulin pump, and automated insulin delivery system education.

Although the dyads reported generally positive experiences with diabetes technology education, both the dyads and the clinicians identified pros and cons to the different training modalities (Figure 1). Although telemedicine education minimized time away from work and school, families and clinicians overwhelmingly preferred in-person education because it allowed for better focus and hands-on troubleshooting when learning to use the devices (Theme 1). One mother reported: “There’s something about being in person that causes me to—like pay more attention. I don’t know, I just like that in-person feeling.” A diabetes educator noted that troubleshooting device placement and connections between devices and smartphones is best done in person: “I think having someone there to physically troubleshoot any little issue that pops up is probably the most valuable for them. I also find that there’s usually less distractions if we have an in-person environment.”

Despite the convenience of telemedicine education, participants identified challenges: Problematic internet connections, difficulties visualizing and hearing the education, and distractions in the home setting made telemedicine education less preferred (Theme 2). When asked about the essential components for a successful telemedicine visit, a clinician listed the following elements: “A good internet connection at home, with data or Wi-Fi. A way to look at the information—are they on a small screen? A big screen makes it a lot easier.” This clinician also acknowledged that there was no previsit screening to determine whether families have these elements present before a telemedicine appointment.

Physical aspects of diabetes technology education are best suited for hands-on, in-person learning, whereas intellectual concepts can be taught virtually (Theme 3). Families and clinicians preferred in-person education because learning to use diabetes technology requires mastery of physical skills, such as placing a pump site and entering insulin delivery settings into the device. A diabetes educator reported:

I feel like the in-person trainings, it’s a lot easier to troubleshoot, problem solve and really help them. . . . [Telemedicine education is] not always that ideal because the lighting’s bad, you can’t see what they’re doing, they’re on a phone device, the phone is staring at the ceiling so you can’t see them. And so, you’re constantly redirecting, hey, can you move yourself into the camera? Where when they’re in person you see it all right in front of you.

Many clinicians acknowledged that telemedicine, EMR messaging, and phone-based communications were helpful in coaching families on more intellectual aspects of pump and CGM use, such as identifying and acting on glycemic patterns.

## Topic Area 2: Information Overload

Although clinicians recognize the importance of delivering structured diabetes technology education, they struggle to balance covering all required topics with families’ individual needs (Theme 4). In reflecting on the challenges of the insulin pump initiation appointment, one mother noted: “It was a lot of information, let’s just start with that. It was a lot—it was a lot of information, and I had reached out a couple times after we got it with like questions.” Clinicians expressed concerns about families’ abilities to process large quantities of information and acknowledged that individuals learn through different modalities and at different rates:

When I think specifically about diabetes technology, which can be very difficult to understand, a lot of numbers and new words involved for families that are just not used to it, I find that they’re just not always ready to absorb the information that we have to present.

Diabetes care and education specialists acknowledged the importance of addressing all topics required by manufacturers for device training; they also recognized the significant intrinsic cognitive load of the material and need to individualize their teaching: “We usually have a 1-month advanced pump class. Okay? That advanced pump class may not end up being an advanced pump class, depending on where the patient is. We might just be reviewing pump safety again.”

Learning to use CGM required families to make emotional adjustments to the sheer number of glucose values they were suddenly able to see, whereas insulin pump therapy posed cognitive challenges (Theme 5). Learning to use CGM centered around greater awareness of glucose levels, as noted by one mother: “So, we kind of all had to learn that, after he eats we’re gonna see highs. So, don’t panic—don’t try to overcorrect those.” Whereas fingerstick blood glucose levels are measured several times throughout the day, CGM provides 288 data points per day. Learning to recognize expected glycemic variations in CGM values, particularly after meals, required families to accept seeing nonactionable higher glucose levels. This sentiment was echoed by a father:

It was a little overwhelming at first, I’m not gonna lie. But I think we—we have a range for him. If he’s at a certain range, he’s okay. So, that kind of I think alleviated some of our anxiety, like we didn’t have to be looking at the thing every 5 minutes. And the alarm for the highs and the alarm for the lows, that was what we were mainly looking for. There’s no alarm, he’s all right.

Although navigating CGM required psychological adjustments, learning to use insulin pumps placed cognitive demands on the families. One mother with more than 6 years of experience as a T1D parent reflected on the overwhelming amount of information that families must learn:

It just scares me because I feel like I went, literally in one day, went from being mom to being—a nurse, a doctor, a mathematician, a nutritionist. You’re wearing all these different hats, which most parents do, in general. But this is the only— and I don’t like to call it disease—this is the only medical condition that I see where the care becomes 100% on the individual and her family. . . . And it’s kind of like your child gets diagnosed with this life-changing condition, and it’s like here’s books and here’s medicine. You’re gonna figure it out.

When asked about adjusting insulin delivery settings, another mother expressed hesitance at taking on additional responsibility for T1D management when initiating an insulin pump:

I know how to read all the data and the reports, at least the ones that are accessible to me. So yeah. I mean, I feel like I know how to do everything. I mean, it’s simple enough. Adding anything extra, I feel like would just be another learning curve. So we already have so many other things that we have to learn and be on top of [with T1D].

## Topic Area 3: The Importance of Experiential Learning

Whereas structured diabetes technology education is often delivered in a discrete number of sessions, dyads reported that real-world experiences support them learning independent self-management skills over time as the information became applicable and relevant (Theme 6). A clinician likened learning to use an insulin pump to the manual that comes with a new car, noting that much of the educational content was irrelevant to until real-world learning opportunities presented themselves:

But until they actually have to do it, they won’t learn it. . . . I think that’s kind of what the learning looks like—all of those bits of logistic learning come at different stages and different places for different families. . . . I think probably the first year that someone is on a pump, they’ve experienced, more than likely, one of everything, whether it’s site issue, or a pump failure or, you know, all of those things that they would need to do a little bit more advance problem-solving through.

Families echoed this sentiment, reflecting on the value of day-to-day experiences using technology in different situations:

I think it’s just doing it and doing it every day with every meal and every single thing he puts into his mouth. So, I think it’s just that confidence of knowing that, okay, we’ve done this before, we’re good with this. I’m still not super confident with sick day—we’ve only had 1. But I think it’s just doing it and the repetition of having to—it’s just kinda become second nature.

In acknowledging different rates of learning and the predetermined number of appointments and time allotted for diabetes technology education, families and clinicians recognized that real-world learning is a crucial source of learning. A diabetes educator noted:

All of that stuff is great information, but for me to sit here with a checklist and go through it in a diabetes education visit when it’s not relevant to them, the information is in one ear and out the other. . . . Our patients have access to us all the time through [the EMR], we’re addressing those things, maybe not in a prescribed curriculum, or a specific visit, but they’re just kind of as those issues arise.

Communicating with families through the EMR or phone calls about failed pump sites, managing lows during exercise, or navigating the first bout of gastroenteritis while using an insulin pump helped the families gain experience and confidence. These communications after the pump starts also allowed clinicians to distribute the information over time in smaller, more manageable bites.

However, even after extended periods of using and applying diabetes technologies, families’ comfort with self-management varies. Although some families progress from pattern recognition to self-management in adjusting AID settings, other families only act on direct recommendations from the clinical team (Theme 7). In explaining her journey toward self-management, one mother explained the value of being willing to experiment:

Just trial and error, really. What worked, what didn’t work, what—yeah. . . . And it would just be kind of trying things and then checking in at our appointments with our providers. Like, hey, we try just doing 10 grams of carbs, fake carbs when she falls asleep. We’re like, cool, right on, that works. Or maybe you could also try increasing the [the insulin delivery at this time]. Okay. We’ll try that too. And things like that. So just time.

As a stepping stone, many families would initially propose their planned changes to their clinical team through EMR messaging, seeking to learn from the team’s feedback. One mother reported: “I contacted endocrinology, and I said, ‘Hey, I want to adjust his background [insulin delivery], what do you think?’ And they were like, yeah, we trust you. And so I did, I adjusted them.” Whereas some families embraced the trial-and-error approach to self-management, others expressed concerns to the clinical team: “I’ve heard families say, ‘I don’t adjust any of my other medications on my own, why am I allowed to adjust my insulin?’” Helping families to reframe insulin as a hormone that the body produces varying amount of in different situations helped some clinicians to support families in self-management, although many families remained hesitant:

I will tell you most of the families I work with are not interested in looking at pump reports. I have some that love data, and they understand the reports. But a lot of them are like, that’s a lot, that’s a lot of information, I don’t even know what to do with that.

## Discussion

Diabetes education is a central pillar of T1D management.^27^ Even with the recommended quarterly clinic appointments, parents and youth are jointly responsible for numerous daily decisions about management and insulin dosing. This study explored the educational experiences of parent-child dyads learning to use CGM and insulin pumps and the clinicians involved in their care and education. Although telemedicine approaches to diabetes technology education reduce time away from work and school, participants preferred in-person education due to poor internet connections, distractions in the home, and small screens that created challenges for teaching the physical skills required to use diabetes technologies. Structured education requires clinicians to address a checklist of topics and is often constrained by a fixed number of sessions; this prescriptive approach often overlooks (1) the cognitive overload and emotional burden related to the amount of information to be learned and (2) variations in individual learning pace and capacity. What cannot be addressed through structured education is supplemented through real-world experiential learning, which can be supported remotely by the clinical team to help families gain confidence. Differences in parents’ willingness to independently adjust pump settings through trial and error led to variations in individuals’ diabetes technology self-management capacity.

Although prior research has shown high satisfaction with the use of telemedicine for diabetes clinic appointments,^28^ the information covered during a typical routine T1D clinic appointment differs greatly from the physical skills that families initiating CGM, insulin pumps, or AID must learn. When telemedicine options are offered to best accommodate individual needs, clinicians in this study recommended advanced screening to ensure that families have a reliable internet connection; a quiet, distraction-free space; and a screen sufficiently large to allow for visualization of the physical skills being taught. Virtual diabetes technology education may be particularly useful for those living in more remote locations father from diabetes centers.^29^ Parents have found telemedicine CGM initiation to be effective^15,30^; however, there is less research about insulin pumps and AID initiation. In a retrospective study of youth initiating insulin pump therapy, there were no significant safety events or differences in A1C at 3 or 6 months after pump initiation.^31^ Telemedicine AID education has also been shown to support equitable device uptake, and remote AID initiation led to comparable glycemic improvements compared to in-person education.^13,32^ However, it is important to note that many of the participants in these studies were already using insulin pump therapy, and so it remains unclear whether telemedicine education is appropriate for those transitioning from multiple daily injection therapy to an insulin pump.

Clinicians and dyads acknowledged the challenge arising from the sheer amount of information that families must process and quickly apply when initiating CGM or insulin pumps. Dyads and clinicians expressed concerns about presenting such complex information within the confines of clinic appointments, suggesting that presenting information in smaller quantities over time (spaced education) may support better learning.^33,34^ Family-initiated EMR and phone-based communication with the clinical team after initiating diabetes technology may help to address real-life challenges and to bridge the gaps of what cannot be effectively addressed during structured education. This approach aligns with experiential learning, wherein a concrete, real-world experience affords learners an opportunity to reflect on the experience, form new ideas and draw conclusions to facilitate a deeper understanding of the experience, and then test the new ideas in a novel situation.^35^ However, relying on experiential learning may also omit crucial concepts, depending on individual real-world T1D experiences.

Not all families progress to independent T1D self-management. To date, there is no direct evidence to support that independent dose adjustments predict better glycemic control; however, studies have demonstrated that greater engagement with insulin pump therapy, including the number of boluses administered and number of insulin delivery settings programmed,^19,36^ predicts better glycemic control. Some families that reported independently adjusting insulin delivery settings expressed comfort with a “trial-and-error” approach to diabetes management, whereas those who preferred to rely on the clinical team were noted to be less interested in independently reviewing their data, possibly due to being overwhelmed with T1D management and/or the perception that adjusting medications is the responsibility of trained medical professionals. Additional studies are needed to determine whether independent adjustments to insulin delivery settings predict superior glycemic control and to understand the characteristics that support families in reaching this level of management.

Despite the lessons learned from these dyads and clinicians, this study is limited by the inclusion of English-speaking dyads receiving care at a single pediatric diabetes center. Institutional practices for CGM, insulin pump, and AID initiation vary according to geographic location and the personnel available to support youth with T1D and their families; as a result, these data are not generalizable to all settings. Including individuals initiating different insulin pumps and AID increased the generalizability of the results; however, it may have led us to overlook the different needs of learners using particular systems. Participants had used CGM and insulin pump therapy for ≥1 month at enrollment, ensuring they had sufficient time to experience the technologies; however, they may not have recalled all details about their experiences initiating these devices.

Diabetes technology has evolved rapidly over the last decade and has redefined the standard of care for youth with T1D. Although CGM and AID can optimize glycemia and reduce the burden of management,^1-3^ optimal use of these systems requires considerable user engagement. Clinicians and dyads both expressed a strong preference for in-person diabetes technology education and articulated the challenges of current educational approaches. With their central role in supporting people with diabetes using CGM and AID, certified diabetes care and education specialists should use their experience to advocate for effective approaches to technology education.^37^ Although experiential learning can address some of the limitations of current approaches to structured education, it may unintentionally omit crucial concepts. Educational strategies are needed to mitigate information overload and to support families in diabetes self-management are needed.
